# Effects of Temperature and Salinity on Perfluorooctane Sulfonate (PFOS) Toxicity in Larval Estuarine Organisms

**DOI:** 10.3390/toxics12040267

**Published:** 2024-04-02

**Authors:** Katy W. Chung, Peter B. Key, Philip Tanabe, Marie E. DeLorenzo

**Affiliations:** National Oceanic and Atmospheric Administration (NOAA), National Ocean Service, National Centers for Coastal Ocean Science, Charleston, SC 29412, USA; pete.key@noaa.gov (P.B.K.); philip.tanabe@noaa.gov (P.T.); marie.delorenzo@noaa.gov (M.E.D.)

**Keywords:** PFOS, fish, crustaceans, gastropods, toxicity, salinity, temperature, multi-stressor

## Abstract

Perfluorooctane sulfonate (PFOS) is a persistent contaminant that has been found globally within the environment. Key data gaps exist in the toxicity of PFOS to marine organisms, especially estuarine species that are crucial to the food web: fish, shrimp, and mollusks. This study developed toxicity thresholds for larval estuarine species, including grass shrimp (*Palaemon pugio*), sheepshead minnows (*Cyprinodon variegatus*), mysids (*Americamysis bahia*), and Eastern mud snails (*Tritia obsoleta*). Multiple abiotic stressors (salinity and temperature) were included as variables in testing the toxicity of PFOS. Acute 96 h toxicity testing under standard test conditions of 25 °C and 20 ppt seawater yielded LC_50_ values of 0.919 mg/L for *C. variegatus*, 1.375 mg/L for *A. bahia*, 1.559 mg/L for *T. obsoleta*, and 2.011 mg/L for *P. pugio*. The effects of increased temperature (32 °C) and decreased salinity (10 ppt) varied with test species. PFOS toxicity for the sheepshead minnows increased with temperature but was not altered by decreased salinity. For grass shrimp and mud snails, PFOS toxicity was greater under lower salinity. The combination of higher temperature and lower salinity was observed to lower the toxicity thresholds for all species. These data demonstrate that expanding toxicity testing to include a wider range of parameters will improve the environmental risk assessment of chemical contaminants, especially for species inhabiting dynamic estuarine ecosystems.

## 1. Introduction

Per- and polyfluoroalkyl substances (PFAS) are a group of fabricated chemicals that have been used in a variety of industries since the 1940s, including food packaging, commercial household products, and electronics manufacturing [1]. In the PFAS group, perfluorooctane sulfonate (PFOS) and perfluorooctanic acid (PFOA) have been the most extensively produced and studied. Both chemicals are very persistent in the environment and have been detected in humans and animal tissues, where they have the ability to accumulate and persist over time [1]. PFAS have been found in drinking water but are typically localized to, and associated with, specific facilities, such as landfills, wastewater treatment plants, and firefighter training facilities [1]. PFOA and PFOS are no longer manufactured in the United States, but they are still produced internationally and can be imported into the United States in consumer goods, such as carpet, leather and apparel, textiles, paper and packaging, coatings, rubber, and plastics [1]. 

PFOS has been identified as one of the most concerning of these chemicals due to its multiple health risks [2], including immunotoxicity, hepatotoxicity, carcinogenicity, and developmental and reproductive effects in humans [3,4,5]. Compared to freshwater fish, less is known regarding the bioeffect thresholds for these compounds in marine and estuarine species. PFAS have been documented in marine and estuarine waters worldwide [2,6,7,8,9]. PFAS levels in edible fish species sampled from Charleston Harbor, SC, USA, were detected (12.7 ng/g wet weight in striped mullet to 85.4 ng/g wet weight in spotted seatrout), and a number of other studies have documented the presence of PFAS levels in estuarine fish [10,11,12,13,14]. PFAS compounds have also been measured in estuarine invertebrates [15,16,17,18]. In a review of data published between 2000 and 2020, PFOS and PFOA were found globally in the seven major oceans, PFOS was the most abundant PFAS compound in sediment, and PFOS was the most common PFAS detected in plankton, fish, reptiles, and birds [19].

Most research on PFOS has dealt with its presence in tissue and water; thus, some understanding of organism exposure exists, yet there are minimal data to assess whether these environmental concentrations pose a risk to organism health. The role of climate change in the toxicity of PFOS has not been adequately explored. The organisms used in the current research were larval grass shrimp (*Palaemon pugio*), sheepshead minnow (*Cyprinodon variegatus*), mysid (*Americamysis bahia*), and mud snail (*Tritia obsoleta*), as sensitive and representative species of estuarine invertebrates and fish. 

Grass shrimp are abundant crustaceans in tidal creeks along the East and Gulf Coasts of the USA, from Maine to Texas, with densities as high as 40 shrimp/m^3^ [20]. Grass shrimp occupy important functions in the estuary, breaking down detritus and recycling nutrients, and serving as a food source for commercially and recreationally important marine organisms [21]. In addition to their abundance and ecological importance, grass shrimp have been shown to be sensitive to chemical contaminants, making them an excellent species for toxicity testing [22,23]. 

The fish species tested was the sheepshead minnow, which is a common inhabitant of the Eastern Atlantic and Gulf Coasts of the USA [24]. Sheepshead minnows are both eurythermal and euryhaline [25,26]. They are an important prey item for larger fish species and are an established toxicity test species [27,28]. 

Mysids are small crustaceans that occur in coastal estuaries from the Gulf of Mexico to Narragansett Bay, RI, USA [29]. Mysids feed on plankton and detritus and are consumed by a variety of estuarine organisms. Mysids have been used extensively in regulatory toxicity testing, since they can be obtained from commercial cultures and are generally considered to be more sensitive than many other test species [29].

The Eastern mud snail is also a common estuarine invertebrate [30]. During their life cycle, they inhabit two important habitats for chemical exposure; the motile larvae are found in the water column, where they feed by filtering, and adult snails are found on the marsh flat, feeding by scraping biofilms from the sediment surface. This activity promotes nutrient regeneration and bioturbation [30,31]. Toxicity testing with the mud snail larval life stage has shown it to be an especially sensitive marine invertebrate [32,33].

While the estuarine species selected for this study are uniquely adapted to changing environmental conditions, they may not be able to withstand the multi-stressor impact of chemical exposure and abiotic stressors. Rates of chemical uptake and metabolism may be altered by temperature and salinity, leading to changes in toxicity thresholds [32]. 

Regulatory guidelines for the protection of marine species are not yet available for PFOS. With this research, we extend our knowledge of PFOS toxicity in four estuarine organisms under multiple temperatures and salinities. The objective of this research was to determine acute toxicity thresholds for representative fish and invertebrate species to inform the establishment of saltwater aquatic life criteria and to assess the role that abiotic stressors, such as temperature and salinity, play on the survival of larval estuarine organisms in co-exposures with PFOS.

## 2. Materials and Methods

### 2.1. Test Organisms

The common holding conditions for all species were 20 ppt 1 µm filtered seawater at a 16L:8D photoperiod and 25 °C. Seawater for all acclimations and bioassays was drawn from Charleston Harbor estuary, SC, USA (N 32°45′11.52″; W 79°53′58.31″), at high tide (±2 h), at salinities between 28 and 35 ppt. It was then allowed to settle for at least 4 d in one of four 284,000 L tanks. The seawater was then pumped into a 19,000 L secondary holding tank where it was polished via sand filtration, UV sterilization, and 5 µm filtration. In the laboratory, this seawater was held in a 600 L tank and treated further with UV sterilization and 1 µm filtration before dilution.

Adult, female, egg-bearing *Palaemon pugio* (grass shrimp) (2–3 cm in length) were obtained from Leadenwah Creek (N 32°38′51.00″; W 80°13′18.05″), a tidal tributary of the North Edisto River, SC, USA (used as a long-term reference site) [34,35]. The reference site sediment and animal tissue concentrations of PFOS were verified to be at background exposure levels. The mean of measured PFOS sediment concentrations from Leadenwah Creek was less than instrument detection limit (MDL = 0.6 ng/g wet weight). Baseline mean shrimp and snail tissue concentrations of PFOS from the site were 1.3 and 1.5 ng/g wet weight, respectively. Grass shrimp were acclimated 7–14 d in 76 L tanks, and the adult shrimp were fed Tetramin^®^ (Tetra, Spectrum Brands Pet, LLC, Blacksburg, VA, USA) fish flakes and newly hatched *Artemia* ad libitum. To acquire larvae for testing, gravid adult shrimp were placed in brooding chambers within 10 L aquaria until the embryos hatched.

Adult *Cyprinodon variegatus* (sheepshead minnows) were obtained from Aquatic BioSystems, Inc. (Fort Collins, CO, USA), and separated by sex in a recirculating living stream. Adult sheeshead minnows were maintained under the IACUC-approved protocol 2023.002. Fish husbandry followed optimized conditions for breeding and rearing of sheepshead minnows [36]. Fish were bred in 76 L tanks with a breeding cage, egg collection tray, and spawning group of 2 males and 4 females. Fish were fed Tetramin^®^ fish flakes twice daily. Spawning occurred overnight, and embryos were collected in the morning. Embryos were checked for viability under a dissecting microscope (SZ61, Olympus, Tokyo, Japan), transferred to glass finger bowls with 20 ppt seawater, aerated, and allowed to hatch.

Adult *Americamysis bahia* (mysid) (7–9 mm in length) were also obtained from Aquatic BioSystems, Inc. (Fort Collins, CO, USA), to serve as brood stock for producing larvae. The adults at a 3:1 ratio (female/male) were placed in 76 L tanks and fed newly hatched *Artemia salina* ad libitum. 

Adult *Tritia obsoleta* (mud snails) (15–18 mm in shell length) were also collected from Leadenwah Creek and acclimated in the laboratory in 76 L tanks at a density of approximately 150 snails per aquarium, and they were fed Tetramin^®^ fish flakes daily ad libitum. To obtain mud snail larvae for testing, egg capsules laid by adult snails on the sides of the aquarium were scraped using a clean razor blade and transferred to a glass finger bowl (1.0 µm filtered 20 ppt aerated seawater), until larvae hatched. 

### 2.2. PFOS Preparations

PFOS (CAS: 1763-23-1, 97% purity, Santa Cruz Biotechnology, Dallas, TX, USA) stock was dissolved in deionized (DI) water in an amber glass container, stirred on a stir plate with a glass stir-bar for 4 h, then stored at room temperature in a dark environment. The 10,000 mg/L stock was quantified as 9487.57 mg/L. Each day, the PFOS stock was stirred for an hour before dosing, and stirring was continued while dosing. To prepare each replicate dose, a graduated cylinder was partly filled with seawater; then, the appropriate volume of PFOS stock was added and the appropriate quantity of DI water was added (to equal 0.1% across all treatments and control). Then, seawater was added to reach the total volume. Every 24 h, new doses were prepared, and the surviving animals were transferred to the new test solutions.

### 2.3. Experimental Conditions

#### 2.3.1. Temperature

All experiments were conducted in environmental chambers (Percival Scientific IntellusUltra C8, Perry, IA, USA) with a photoperiod of 16 h L/8 h D. Two temperatures were selected for testing: a standard bioassay temperature of 25 °C, and 32 °C, which represents the upper range of temperature found in southeastern USA estuarine habitats [32,37]. Temperature was controlled using the environmental chambers and varied within ±2 °C. 

#### 2.3.2. Salinity

The salinity conditions selected for testing were 10 and 20 ppt. The 20 ppt salinity corresponds with the standard toxicity test conditions for these estuarine species, while the 10 ppt salinity is typical of low salinity found in southeastern USA tidal habitats [37]. Full-strength seawater was diluted with deionized water to achieve the exposure salinities and filtered to 1.0 µm for testing. 

#### 2.3.3. Acclimation

Before exposures occurred, all newly hatched larvae were maintained in 20 ppt seawater in a 25 °C incubator under fluorescent lighting (standard conditions) and then acclimated to the appropriate multi-stressor conditions [32]. Salinity changes did not exceed 5 ppt per 90 min, and temperature was adjusted approximately two degrees per 90 min. For the full-factorial design, larvae were split into 4 bowls with filtered 20 ppt seawater. Half of the larvae were kept in the 25 °C incubator for 90 min. The other half of the larvae were acclimated in a 32 °C incubator for 90 min. One bowl in each incubator remained at 20 ppt, and these larvae were handled the same way as those receiving the salinity acclimation. The 20 ppt seawater in both 25 °C incubator and 32 °C incubator was then diluted to 15 ppt, and the larvae were acclimated for another 90 min. The 15 ppt seawater in both incubators was further diluted to 10 ppt, and the larvae were acclimated for another 90 min. Larvae were not fed during the acclimation period. 

#### 2.3.4. Testing

Range finder tests were conducted to establish the concentration ranges used for all four larval species (i.e., concentrations were selected to bracket the median lethal effect concentration). All exposures were 96 h static renewals with larval animals (24–48 h post hatch) fed newly hatched *Artemia salina* ad libitum during testing. Range finder tests were conducted to establish the concentration ranges used for all four larval species. Larval survival in each dish was assessed at 24 h, 48 h, 72 h, and 96 h. Daily test renewals were performed, and water quality (temperature, dissolved oxygen, salinity, and pH) was measured. Grass shrimp, sheepshead minnow, and mysid toxicity tests were based on standard methods by ASTM E729-96 (2014c) [38]. Mud snail toxicity tests were based on methods of ASTM E729-96 (2014c) and DeLorenzo et al. [32,38].

To assess the effects of salinity and temperature on PFOS toxicity, five concentrations of PFOS were tested, plus a control, with two salinity levels (10, 20-ppt) and two temperature levels (25 °C, 32 °C). The larval organisms were tested in glass dishes (230 mL for fish, shrimp, and mysids and 50 mL for snails) covered with a crystal-grade polystyrene Falcon Petri dish to prevent evaporation. Each treatment had three replicates with 10 larvae per chamber (larval snails had 20 per replicate). Nominal treatment concentrations were 0, 0.21, 0.62, 1.85, 5.56, and 16.67 mg/L for mysids; 0, 0.63, 1.25, 2.5, 5.0, and 10 mg/L for snails; 0, 0.1, 0.31, 0.93, 2.78, and 8.33 mg/L for the sheepshead minnows; and 0, 0.63, 1.85, 5.56, 16.7, and 50 mg/L for grass shrimp (Tables S2–S5). The range of selected test concentrations is generally higher than those reported in surface waters, but this allowed for the determination of median lethal thresholds.

### 2.4. Chemical Analysis

Water samples were collected at 0 h (immediately after dosing) and 24 h (before treatment renewal) of exposure from each treatment and stored at −20 °C in a dark environment until analysis. All samples were analyzed using an Agilent Infinity II (Agilent, Santa Clara, CA, USA) liquid chromatography instrument attached to a SCIEX Triple Quad 5500+ LC-MS/MS (SCIEX, Framingham, MA, USA). A Zorbax Diol (4.6 mm ID, 12.5 mm, 6 μm particle size) attached to an Agilent InfinityLab Poroshell 120 EC-C18 column (4.6 mm ID, 100 mm, 2.7 μm particle size) was used for separation of PFOS and each sample run with a ramping LC solvent gradient with methanol and nanopure water, each containing 20 mmol/L ammonium acetate. Two multiple reaction monitoring (MRM) transitions were employed for each PFOS, one for quantitation and the other for confirmation of the PFOS. Measured PFOS treatment concentrations are available in the Supplementary Materials (Tables S2–S5). 

### 2.5. Statistical Methods

Lethal concentration (LC) toxicity thresholds (96 h LC_50_ values) with 95% confidence intervals (CI) were determined using Statistical Analysis Software (SAS) probit analysis (PROC PROBIT, SAS V.9.4, Cary, NC, USA) using the measured (T = 0 h) chemistry concentrations (Tables S2–S5). The LC_50_ ratio test was used to test for significant differences between LC_50_ values for standard conditions and climate stress conditions [39]. To further assess toxicity of PFOS, Lowest Observed Effect Concentration (LOEC) and the No Observed Effect Concentration (NOEC) were determined by ANOVA followed by Dunnett’s procedure for comparison to the control response. The LOEC was the lowest tested concentration, significantly different from control. The NOEC was the highest tested concentration tested, which was not statistically significant from control. A series of three-factor ANOVAs with replication was performed (PROC MIXED in SAS 9.4) to examine differences in mortality of fish, shrimp, mysids, and snails using a maximum-likelihood-based (method = REML) approach with standard covariance structures (type = VC). The three factors (PFOS concentration, temperature, and salinity) were set as fixed-effect factors, and replicate was set as a random-effect factor. Post hoc comparisons were performed using the LSMeans statement with the ADJUST = DUNNETT option for PFOS comparisons versus control levels. The SLICE statement was used to examine for partitioned effects within significant interaction terms.

## 3. Results

### 3.1. Water Quality and Chemisty

Water quality measurements taken for standard testing (25 °C, 20-ppt) all fell within the thresholds established by ASTM method E 1241-98 [40]: temperature (25 °C ± 2), DO (>4.00 mg/L), salinity (20 ppt ± 1), and pH (7.80 ± 1.00) (Table S1). The high-temperature (32 °C) tests had a measured range 32.18–33.10 °C for the four species tested (Table S1). The standard temperature (25 °C) tests had a measured range 24.10–25.58 °C for the four species tested (Table S1). Measured values were all within ±1 °C. Salinities measured were all within ±1 ppt of the targeted 10 ppt (9.36–10.40 ppt) and 20 ppt (19.75–20.57 ppt) (Table S1).

PFOS values from T = 0 and 24 h samples were measured and are reported in Tables S2–S5 for the four species. There was minimal change in PFOS concentration from T = 0 to T = 24 h; therefore, the T = 0 measured concentrations were used to calculate the LC50 values. There was noted variability in the agreement between nominal and measured concentrations across treatments, which may relate to loss of the compound through binding to the glass test chamber (Tables S2–S5).

### 3.2. Species Sensitivity under Standard Testing Conditions

Control survival was 100% for the fish and mysids, and >90% for grass shrimp and snails for all the temperature and salinity tests. Under standard testing conditions, larval sheepshead minnows were the most sensitive species tested, followed by mysids, mud snails, and grass shrimp. The 96 h LC_50_ values for measured PFOS tested under standard conditions (25 °C, 20 ppt) were 0.919 mg/L for fish, 1.375 for mysids, 1.559 for mud snails, and 2.011 mg/L for grass shrimp (Table 1). The NOEC and LOEC values under standard testing conditions were 0.30 mg/L NOEC and 0.70 mg/L LOEC for fish, 0.80 mg/L NOEC and 2.06 mg/L LOEC for mysids, <0.88 mg/L NOEC and 0.88 mg/L LOEC for snails, and 0.81 mg/L NOEC and 2.18 mg/L LOEC for shrimp.

### 3.3. Effect of Salinity

Decreasing salinity generally increased sensitivity to PFOS for the invertebrate species tested but did not affect sensitivity for the fish. Compared to standard test conditions (25 °C, 20-ppt), lower salinity (25 °C, 10-ppt) significantly increased the toxicity of PFOS to mud snails, with 96 h LC_50_ values of 1.559 mg/L at 25 °C, 20-ppt, and 0.144 mg/L at 25 °C, 10 ppt salinity (LC_50_ ratio test, *p* < 0.0001) (Table 1). While the LC50 values were not significantly different at lower salinity for grass shrimp and mysids, the No Observable Effect Concentration (NOEC) values were lower at the lower test salinity, decreasing from 0.80 mg/L to 0.26 mg/L for mysids and from 0.81 mg/L to <0.53 mg/L, respectively. Fish, however, were less sensitive to lower salinity, with little change in NOEC (0.30 mg/L at 20 ppt and 0.28 mg/L at 10 ppt). 

### 3.4. Effect of Temperature

Compared to standard test conditions (25 °C, 20-ppt), higher temperature (32 °C, 20-ppt) generally increased the toxicity of PFOS to all species tested, with mean 96 h LC_50_ values of 0.344 mg/L for sheepshead minnows, 1.077 mg/L for mysids, 1.297 mg/L for mud snails, and 1.652 mg/L PFOS for grass shrimp (Table 1). The effect of temperature was greatest for fish, with a 2.67-fold increase in sensitivity at higher temperature. Fish NOEC values decreased from 0.30 mg/L (25 °C, 20 ppt) to 0.18 mg/L (32 °C, 20 ppt), and the LOEC decreased from 0.70 mg/L to 0.28 mg/L. Mysid sensitivity also increased at the higher temperature, with the NOEC value decreasing from 0.80 mg/L (25 °C, 20 ppt) to 0.19 mg/L (25 °C, 10 ppt) and the LOEC decreasing from 2.09 mg/L to 0.91 mg/L.

### 3.5. Temperature, Salinity, and PFOS Interactions

**Figure 1:**
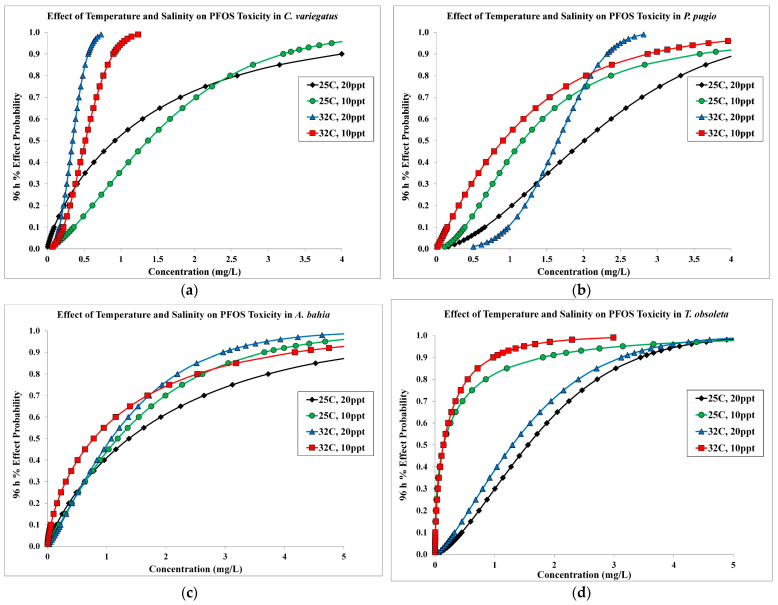
Predicted 96 h percent effect probability curves from SAS probit analysis for each exposure condition. (**a**) = sheepshead minnow (*C. variegatus*), (**b**) = grass shrimp (*P. pugio*), (**c**) = mysid (*A. bahia*), (**d**) = mud snail (*T. obsoleta*).

Under combined higher temperature (32 °C) and lower salinity (10 ppt) conditions, larval snails (LC_50_ = 0.144 mg/L) were the most sensitive species tested followed by fish (LC_50_ = 0.518 mg/L), mysids (LC_50_ = 0.780 mg/L), and shrimp (LC_50_ = 0.904 mg/L) (Table 1). The predicted percent effect probability curves from the probit analysis demonstrate that the combination of increased temperature and lower salinity generally increases sensitivity to PFOS, but responses to abiotic stressors varied by species (Figure 1). The fish species tested was most sensitive to PFOS at higher temperature, while toxicity was not influenced by a lower salinity exposure (Figure 1a). The grass shrimp and mud snail were more sensitive to PFOS at lower salinity, but toxicity was less altered by higher temperature (Figure 1b,d). With mysids, toxicity to PFOS did increase under the higher-temperature and lower-salinity conditions, but the effects were more subtle and did not substantially shift the probability effects curve (Figure 1c). 

Multifactor analysis of variance revealed interactions between chemical and abiotic stressors (Table S6). For grass shrimp larvae, statistical analysis confirmed that PFOS concentration (*p* < 0.001), temperature (*p* = 0.0181), and salinity (*p* < 0.0001) all had a significant effect on mortality (Table S6A). However, only the interaction of PFOS × salinity was significant (*p* < 0.0001) (Table S6A). Post hoc tests revealed at which concentrations the interaction occurred. There were significant differences in mortality between the 10 and 20 ppt salinities at the three lowest PFOS nominal concentrations of 0.62 (*p* < 0.0001), 1.85 (*p* < 0.0001), and 5.56 mg/L (*p* < 0.0001) (Table S6A). There was no significant interaction between PFOS and salinity at the two highest concentrations, indicating that PFOS alone drove mortality. There were also no significant interactions between PFOS × temperature × salinity and between temperature × salinity.

With the sheepshead minnow larvae, PFOS concentration, temperature, and salinity all had a significant effect on mortality (Table S6B) (*p* < 0.0001). The interaction of PFOS × temperature (*p* < 0.0001) and PFOS × salinity (*p* = 0.0273) also had a significant effect on the mortality of the fish larvae (Table S6B). Further analysis with post hoc tests revealed at which concentrations the interaction occurred (Table S6B). There were no significant differences between the two temperatures at the highest PFOS concentration, indicating that PFOS alone was responsible for mortality (Table S6B). There were significant differences in mortality between the 10 and 20 ppt salinities at PFOS nominal concentrations of 0.31 (*p* = 0.0119), 0.93 (*p* = 0.0001), and 2.78 mg/L (*p* = 0.0420) (Table S6B). As with temperature, there was no significant interaction between PFOS and salinity at the highest concentration. There were also no significant interactions between PFOS × temperature × salinity and between temperature × salinity.

There was no significant interaction between temperature and salinity with PFOS in the exposures to mysid larvae. The only significant effect on mortality was due to PFOS alone (*p* < 0.0001) (Table S6C). This indicates that neither temperature nor salinity had a significant effect on larval mysid mortality. There were also no significant interactions between PFOS × temperature × salinity and temperature × salinity. 

In the exposures with snail larvae, statistical analysis confirmed that PFOS concentration (*p* < 0.0001), temperature (*p* = 0.0001), and salinity (*p* < 0.0001) all had a significant effect on mortality (Table S6D). The interaction of PFOS × temperature (*p* = 0.0021) and PFOS × salinity (*p* < 0.0001) also had a significant effect on the mortality of larvae (Table S6D). Post hoc tests revealed at which concentrations the interaction occurred. There was a significant difference in mortality between the 32 °C and 25 °C temperatures at the three lowest PFOS nominal concentrations of 0.62 (*p* = 0.0004), 1.25 (*p* < 0.0001), and 2.5 mg/L (*p* = 0.0239) (Table S6D). There were no significant differences between the two temperatures at the two highest PFOS concentrations, indicating that PFOS alone was responsible for mortality. There were significant differences in mortality between the 10 and 20 ppt salinities at the PFOS nominal concentrations of 0.62 (*p* < 0.0001), 1.25 (*p* < 0.0001), 2.5 (*p* < 0.0001), and 5.0 mg/L (*p* < 0.0001) (Table S6D). There was no significant interaction between PFOS and salinity at the two highest concentrations, indicating that PFOS alone drove mortality. There were also no significant interactions between PFOS × temperature × salinity and temperature × salinity.

## 4. Discussion

This research demonstrated the importance of not only testing different trophic levels of estuarine organisms to the same contaminant but also of testing under different environmental conditions. Larval life stages were used in this study because they often represent the most sensitive life stage to contaminant exposure. This is true for PFOS toxicity, as previous studies have reported higher LC50 values for adult *C. variegatus* of >15 mg/L [40] and adult *P. pugio* of 9.94 mg/L [41]. The four estuarine species selected successfully inhabit areas with widely variable environmental conditions [21,42,43,44]. Depending on precipitation, season, and tidal cycle, these estuarine organisms experience salinity ranges from 0 to 36 ppt and temperature ranges from 2 to 37 °C [34]. Grass shrimp can tolerate salinities from nearly fresh to full-strength (approximately 33–38 ppt) seawater, but those collected at lower salinities are smaller in size, and larval development is reduced at <10 ppt compared to those from higher salinities [21,34]. While adult grass shrimp are adept osmoregulators, lower salinities negatively affect larval survival, growth, and development and may lead to enhanced chemical uptake [23,45]. Adult and larval Eastern mud snails tolerate salinities ranging from approximately 10 ppt to full-strength (approximately 33–38 ppt) seawater [46]. Lower salinity levels have been shown to inhibit shell growth rate, reduce swimming velocity, prevent metamorphosis, and reduce clearance rate [47]. Sheepshead minnows are also very tolerant of low salinity and high temperatures [26,48]. Mysids can tolerate salinity ranges from 2 to 33 ppt and temperatures up to 34 °C [49,50]. 

Chemical contaminants add another factor to stressors that organisms face in the estuarine world. Whether through runoff, aerial drift, wastewater, or other anthropogenic routes, chemical exposure to these larval organisms can be exacerbated by variations in abiotic factors such as salinity and temperature. 

Temperature has been shown to alter the toxicity of many classes of chemical contaminants, such as pesticides (chlorothalonil and Scourge^®^), which were more toxic to larval grass shrimp at higher temperatures [34]. Similarly, increased temperature (32 °C) resulted in increased toxicity of crude oil to larval shrimp (*P. pugio*), snails (*T. obsoleta*), and fish (*C. variegatus*) compared to 25 °C [32]. Previous PFOS studies with 3-day-old mysids (*A. bahia*) determined 96 h LC_50_ values ranging from 3.6 mg/L to 5.1 mg/L when tested at 20 °C [51,52]. The current study showed that increasing the test temperature resulted in increased mysid sensitivity to PFOS (96 h LC_50_ of 1.375 mg/L at 25 °C and 1.077 mg/L at 32 °C). 

Salinity can also influence the toxicity of chemical contaminants. Available studies suggest that salinity regularly alters the toxicity of other contaminants, including metals, PAHs, and the herbicide atrazine [53,54]. Past study has shown that larval mud snails were more sensitive to oil dispersants (Finasol^®^ OSR 52 and Corexit™ EC9500A) at lower salinities compared to standard testing conditions of 20 ppt seawater [46]. Conversely, the fish *Menidia beryllina* was more sensitive when exposed to the pesticide triadimefon at a higher salinity of 15 ppt (0.218 mg/L) than at 5 ppt (2.74 mg/L) [55]. Hutton et al. tested six other pesticides (bifenthrin, chlorpyrifos, dicloran, paraquat, penconazole, myclobutanil) with *M. beryllina* at two salinities (5 and 15 ppt), with all LC_50_ values lower at higher salinity [55]. Salinity effects on chemical contaminant toxicity vary with the species, life stage, and contaminant tested. The resulting increase in toxicity seen with the larval invertebrates in this study at lower salinity could be due to a combination of osmoregulatory stress and greater bioavailability of the PFOS compound at lower salinities, although no consistent relationship between measured PFOS concentration and salinity was found. Thus, it is likely physiological stress leading to greater water and contaminant intake that increased toxicity.

The mechanics of PFOS toxicity are currently being studied in aquatic organisms, particularly in fish [56,57,58], but the exact route of toxicity is still unknown. PFOS may be involved in reactive oxygen species induction, leading to oxidative stress [57]. How this plays out with the added stresses of temperature and salinity remains to be seen with additional studies.

To better understand how real-world conditions affect toxicity, it is recommended to incorporate a multi-stressor approach to toxicity testing. Current chemical risk assessments use standardized testing conditions that only take into account optimal light, salinity, and temperature for the test species. By looking at how varying abiotic factors affect the species’ response to the chemical contaminant, we can refine the accuracy of the chemical risk assessment. The multi-factor analysis in this study showed that PFOS, temperature, and salinity all had significant effects on mortality in larval sheepshead minnows, grass shrimp, and snails. For larval mysids, only the PFOS exposure levels were driving mortality, indicating that high temperatures and low salinities may be more tolerable. The lack of an interactive effect of temperature × salinity alone for any of the four species selected indicates the ability of these estuarine species to tolerate swings in temperature and salinity. While shifts in climate may increase temperatures and lower salinities, it is the addition of contaminants that can increase the stress in estuarine organisms, leading to mortality.

## 5. Conclusions

Various exposure conditions other than standard laboratory conditions were used to determine possible effects of climate change scenarios. Statistical analysis showed that temperature and/or salinity can be a driver of PFOS in toxicity at all but the highest concentrations for fish, shrimp, and snails. The mysid results showed their resilience to temperature and salinity changes but not to PFOS exposures. 

The results of the LC_50_, LOEC, and NOEC values show that these four species would be protected by the proposed EPA saltwater benchmark for PFOS (0.55 mg/L), although not adequately under high-temperature and low-salinity conditions. The LC_50_ value for sheepshead minnows (0.518 mg/L) would be slightly below the benchmark at temperatures of 32 °C, and the LC_50_ value for mud snails (0.144 mg/L) would be well below the benchmark at salinities of 10 ppt. The draft EPA guidelines include only a modeled acute water column benchmark for marine/estuarine waters due to the lack of supporting toxicity data. The acute value is designed to be protective for short-term (1 h) exposures, so this study’s exposure time of 96 h would be more relatable to the chronic water column criteria, which was designed to be protective of a 4-day average concentration. While not directly comparable, the effects thresholds derived for larval fish and invertebrates in this study may inform future regulatory decisions. Establishing the chemical effects on estuarine organisms under multiple environmental variables will benefit the management and conservation of these sensitive habitats.

## Figures and Tables

**Table 1:** LC_50_ (96 h) values with 95% confidence intervals in parentheses for larval estuarine organisms exposed to PFOS under each test condition. LC_50_s calculated using measured PFOS values (mg/L), SAS probit analysis. The standard test condition is 25 °C, 20 ppt salinity. Asterisk (*) indicates that the LC_50_s are statistically different from each species standard test conditions (LC_50_ ratio test) [39].

	25 °C 10-ppt	25 °C 20-ppt	32 °C 10-ppt	32 °C 20-ppt
Shrimp (*P. pugio*)	1.174 (0.594–2.138)	2.011 (1.577–2.468)	0.904 (0.481–1.229)	1.652 (1.402–1.860)
Fish (*C. variegatus*)	1.368 (1.066–1.749)	0.919 (0.527–1.447)	0.518 (0.430–0.663)	0.344 (0.291–0.397)
Mysid (*A. bahia*)	1.186 (0.827–1.557)	1.375 (0.549–2.305)	0.780 (0.440–1.138)	1.077 (0.776–1.399)
Snail (*T. obsoleta*)	0.144 * (0.019–0.334)	1.559 (1.315–1.761)	0.144 * (0.016–0.306)	1.297 (0.978–1.519)

## Data Availability

The data presented in this study are available on request from the corresponding author.

## Supplementary Materials

The following supporting information can be downloaded at: https://www.mdpi.com/article/10.3390/toxics12040267/s1, Table S1: Water quality measurements from temperature, ad salinity PFOS testing with the four larval species. Mean values are presented with the range in parentheses; Table S2: Mean measured (mg/L) concentrations from the PFOS exposures (T = 0 h and T = 24 h) and % survival for larval grass shrimp (*Palaemon pugio*); Table S3: Mean measured (mg/L) concentrations from the PFOS exposures (T = 0 h and T = 24 h) and % survival for larval sheepshead minnow (*Cyprinodon variegatus*); Table S4: Mean measured (mg/L) concentrations from the PFOS exposures (T = 0 h and T = 24 h) and % survival for larval mysid (*Americamysis bahia*); Table S5: Mean measured (mg/L) concentrations from the PFOS exposures (T = 0 h and T = 24 h) and % survival for larval mud snail (*Tritia obsoleta*); Table S6: Results of three-factor ANOVAs with shrimp (A), fish (B), mysids (C), and snails (D). Interaction effects are the results of PFOS concentration (nominal values), temperature, and salinity on mortality along with any significant interaction between the three factors. Only significant results are listed (*p* value < 0.05). Concentration effects resulted from post-hoc comparisons looking at effects of PFOS interactions with temperature and salinity on mortality as compared to controls. A *p*-value < 0.05 indicates significant differences in mortality occurred with temperature and/or salinity at that concentration. Only significant results are listed.
