# Supplementary material for: Effects of Temperature and Salinity on Perfluorooctane Sulfonate (PFOS) Toxicity in Larval Estuarine Organisms
Source: Toxics. 2024 Apr 2;12(4):267. doi: 10.3390/toxics12040267 (PMC11053673; doi:10.3390/toxics12040267)
Supplement: Supplementary file 1 [file toxics-12-00267-s001.zip › toxics-2920964-supplementary.pdf]

Title: Effects of temperature and salinity on Perfluorooctane Sulfonate (PFOS) toxicity in larval estuarine organisms

Authors and Affiliations:
Katy W. Chung[1*], Peter B. Key[1], Philip Tanabe[1], Marie E. DeLorenzo[1]

[1]National Oceanic and Atmospheric Administration (NOAA), National Ocean Service, National Centers for Coastal Ocean Science, Charleston, SC, USA

*Corresponding author:

Katy W. Chung
NOAA, NOS, National Centers for Coastal Ocean Science
331 Fort Johnson Rd.
Charleston, SC 29412
katy.chung@noaa.gov
843-580-9820

Table S1. Water quality measurements from temperature, ad salinity PFOS testing with the four larval species. Mean values are presented with the range in parentheses.

Parameter	Shrimp (*P. pugio*)	Fish (*C. variegatus*)	Mysid (*A. bahia*)	Snail (*T. obsoleta*)	Exposure
Temperature (°C)	25.50 (24.90 – 26.10)	24.20 (23.80 – 24.70)	25.06 (24.30 – 25.80)	24.16 (23.00 – 25.70)	25°C 20-ppt
	25.58 (25.20 – 25.90)	24.15 (23.70 – 24.90)	24.72 (23.60 – 25.60)	24.10 (23.00 – 26.20)	25°C 10-ppt
	33.06 (32.90 – 33.20)	32.18 (30.00 – 33.10)	33.10 (33.10 – 33.10)	32.96 (32.70 – 33.20)	32°C 20-ppt
	33.06 (32.90 – 33.20)	32.18 (30.00 – 33.10)	33.10 (33.10 – 33.10)	32.96 (32.70 – 33.20)	32°C 10-ppt
Dissolved Oxygen (mg/L)	6.78 (6.22 – 7.70)	6.95 (6.79 – 7.11)	6.49 (5.92 – 6.68)	6.82 (6.52 – 7.16)	25°C 20-ppt
	7.19 (6.65 – 8.13)	7.36 (7.10 – 7.55)	6.67 (6.07 – 7.24)	7.15 (6.83 – 7.58)	25°C 10-ppt
	6.35 (5.64 – 7.58)	6.09 (5.92 – 6.31)	5.48 (4.81 – 5.72)	6.31 (5.86 – 6.68)	32°C 20-ppt
	6.71 (6.01 – 8.14)	6.34 (6.19 – 6.53	5.85 (5.36 – 6.08)	6.43 (6.19 – 6.65)	32°C 10-ppt
Salinity (ppt)	20.48 (20.20 – 20.73)	20.18 (19.90 – 20.38)	20.28 (20.25 – 20.32)	20.56 (19.96 – 21.50)	25°C 20-ppt
	10.40 (10.30 – 10.55)	10.39 (10.26 – 10.50)	10.33 (10.00 – 10.78)	10.05 (10.00 – 10.20)	25°C 10-ppt
	20.02 (19.74 – 20.25)	20.37 (20.19 – 20.60)	20.37 (20.22 – 20.56)	20.40 (20.00 – 21.60)	32°C 20-ppt
	10.20 (10.11 – 10.37)	10.37 (10.23 – 10.49)	9.36 (9.01 – 10.20)	10.25 (10.00 – 11.00)	32°C 10-ppt
pH	7.84 (7.79 – 7.88)	7.88 (7.80 – 7.95)	7.86 (7.82 – 7.90)	8.01 (7.90 – 8.28)	25°C 20-ppt
	7.84 (7.79 – 7.88)	7.99 (7.97 – 8.05)	7.67 (7.46 – 8.00)	7.81 (7.75 – 7.92)	25°C 10-ppt
	7.87 (7.59 – 7.98)	7.90 (7.81 – 8.01)	7.80 (7.70 – 7.87)	8.00 (7.95 – 8.05)	32°C 20-ppt
	7.70 (7.64 – 7.75)	7.90 (7.87 – 7.95)	7.49 (7.42 – 7.56)	7.85 (7.81 – 7.93)	32°C 10-ppt

Table S2: Mean measured (mg/L) concentrations from the PFOS exposures (T=0h and T=24h) and % survival for larval grass shrimp (*Palaemon pugio*).

Temperature (°C)	Salinity (ppt)	Nominal (mg/L)	Measured T=0h	Measured T=24h	% Survival
25	20	0.00	0.00	0.00	100
		0.63	0.36	0.38	96.67
		1.85	0.81	1.09	86.67
		5.56	2.18	2.02	43.33
		16.67	5.3	4.16	3.33
		50.0	32.2	21.88	0.00
25	10	0.00	0.00	0.00	90.00
		0.63	0.53	0.57	76.67
		1.85	1.11	1.35	66.67
		5.56	3.25	3.67	3.33
		16.67	7.73	5.53	0.00
		50	22.91	27.39	0.00
32	20	0.00	0.00	0.00	100
		0.63	0.5	0.47	100
		1.85	0.88	1.34	96.67
		5.56	2.08	2.81	20.00
		16.67	5.37	7.73	0.00
		50	21.84	35	0.00
32	10	0.00	0.00	0.00	96.67
		0.63	0.94	0.65	46.67
		1.85	1.35	1.38	36.67
		5.56	3.42	3.68	6.67
		16.67	7.57	7.96	0
		50	26.84	39.52	0

Table S3: Mean measured (mg/L) concentrations from the PFOS exposures (T=0h and T=24h) and % survival for larval sheepshead minnow (*Cyprinodon variegatus*).

Temperature (°C)	Salinity (ppt)	Nominal (mg/L)	Measured T=0h	Measured T=24h	% Survival
25	20	0.00	0.00	0.00	100
		0.1	0.21	0.17	76.67
		0.31	0.3	0.27	76.67
		0.93	0.7	0.54	63.33
		2.78	1.73	1.48	30.00
		8.33	6.16	4.1	3.33
25	10	0.00	0.00	0.00	100
		0.1	0.18	0.18	90.00
		0.31	0.28	0.29	93.33
		0.93	0.55	0.61	83.33
		2.78	1.49	1.6	56.67
		8.33	4.32	5.23	0.00
32	20	0.00	0.00	0.00	100
		0.1	0.18	0.19	80.00
		0.31	0.28	0.26	76.67
		0.93	0.57	0.5	6.67
		2.78	1.39	1.46	0.00
		8.33	4.91	4.55	0.00
32	10	0.00	0.00	0.00	100
		0.1	0.18	0.18	86.67
		0.31	0.28	0.29	93.33
		0.93	0.57	0.58	40.00
		2.78	1.61	1.54	0.000
		8.33	6.32	1.14	0.00

Table S4: Mean measured (mg/L) concentrations from the PFOS exposures (T=0h and T=24h) and % survival for larval mysid (*Americamysis bahia*).

Temperature (°C)	Salinity (ppt)	Nominal (mg/L)	Measured T=0h	Measured T=24h	% Survival
25	20	0.00	0.00	0.00	100
		0.21	0.24	0.24	73.33
		0.62	0.8	0.73	83.33
		1.85	2.06	1.98	46.67
		5.56	3.68	3.56	3.33
		16.67	8.6	5.15	6.67
25	10	0.00	0.00	0.00	100
		0.21	0.26	0.31	83.33
		0.62	0.93	1.31	66.67
		1.85	1.98	2.39	26.67
		5.56	5.24	6.36	3.33
		16.67	17.29	8.06	0.00
32	20	0.00	0.00	0.00	100
		0.21	0.19	0.19	86.67
		0.62	0.91	0.88	63.33
		1.85	1.61	1.76	33.33
		5.56	5.51	3.71	0.00
		16.67	7.57	8.34	0.00
32	10	0.00	0.00	0.00	100
		0.21	0.24	0.28	66.67
		0.62	0.98	0.9	50.00
		1.85	1.78	2.27	33.33
		5.56	6.22	7.13	3.33
		16.67	11.98	11.47	0.00

Table S5: Mean measured (mg/L) concentrations from the PFOS exposures (T=0h and T=24h) and % survival for larval mud snail (*Tritia obsoleta*).

Temperature (°C)	Salinity (ppt)	Nominal (mg/L)	Measured T=0h	Measured T=24h	% Survival
25	20	0.00	0.00	0.00	100
		0.63	0.88	1.02	66.67
		1.25	1.71	2.24	51.67
		2.5	2.23	3.05	36.67
		5	2.67	3.78	18.33
		10	5.17	3.74	0.00
25	10	0.00	0.00	0.00	91.67
		0.63	0.62	0.7	21.67
		1.25	1.92	2.62	15.00
		2.5	2.55	3.32	5.00
		5	5.72	4.99	1.67
		10	10.04	6.01	0.00
32	20	0.00	0.00	0.00	95.0
		0.63	1.21	1.23	50.00
		1.25	1.62	1.82	35.00
		2.5	2.6	2.94	28.33
		5	2.35	3.55	23.33
		10	3.53	5.02	0.000
32	10	0.00	0.00	0.00	95.0
		0.63	0.6	0.72	16.67
		1.25	1.92	2.44	6.67
		2.5	2.47	3.74	0.00
		5	3.68	5.21	0.00
		10	8.37	6.69	0.00

Table S6. Results of three-factor ANOVAs with shrimp (A), fish (B), mysids(C), and snails (D). Interaction effects are the results of PFOS concentration (nominal values), temperature, and salinity on mortality along with any significant interaction between the three factors. Only significant results are listed (p value < 0.05). Concentration effects resulted from post-hoc comparisons looking at effects of PFOS interactions with temperature and salinity on mortality as compared to controls. A p-value <0.05 indicates significant differences in mortality occurred with temperature and/or salinity at that concentration. Only significant results are listed.

A. Shrimp (*P. pugio*)	
Interaction Effects	*p value*
PFOS	<0.0001
Temperature	0.0181
Salinity	<0.0001
PFOS*Salinity	<0.0001
Concentration Effects	*p value*
PFOS*Salinity 0.62 mg/L	<0.0001
PFOS*Salinity 1.85 mg/L	<0.0001
PFOS*Salinity 5.56mg/L	<0.0001

B. Fish (*C. variegatus*)	
Interaction Effects	*p value*
PFOS	<0.0001
Temperature	<0.0001
Salinity	0.0001
PFOS*Temperature	<0.0001
PFOS*Salinity	0.0273
Concentration Effects	*p value*
PFOS*Temperature 0.93 mg/L	<0.0001
PFOS*Temperature 2.78 mg/L	<0.0001
PFOS*Salinity 0.31 mg/L	0.0119
PFOS*Salinity 0.93 mg/L	0.0001
PFOS*Salinity 2.78 mg/L	0.0420

C. Mysid (*A. bahia*)	
Interaction Effects	*p value*
PFOS	<0.0001

D. Snail (*T. obsoleta*)	
Interaction Effects	*p value*
PFOS	<0.0001
Temperature	0.0001
Salinity	<0.0001
PFOS*Temperature	0.0021
PFOS*Salinity	<0.0001
Concentration Effects	*p value*
PFOS*Temperature 0.62 mg/L	0.0004
PFOS*Temperature 1.25 mg/L	<0.0001
PFOS*Temperature 2.50 mg/L	0.0239
PFOS*Salinity 0.62 mg/L	<0.0001
PFOS*Salinity 1.25 mg/L	<0.0001
PFOS*Salinity 2.50 mg/L	<0.0001
PFOS*Salinity 5.00 mg/L	<0.0001
